# Exploring the Relationship Between Helicobacter pylori Infection and Biliary Diseases: A Comprehensive Analysis Using the United States National Inpatient Sample (2016-2020)

**DOI:** 10.7759/cureus.61238

**Published:** 2024-05-28

**Authors:** Syed O Ahmad, Mohammad AlAmr, Abdulrahman Taftafa, Asim M AlMazmomy, Nourah Alkahmous, Abdullah M Alharran, Abdulhadi M Almarri, Fajer Alyaqout, Abdulbadih R Saad, Abdulaziz M Alazmi, Yousef M Alharran, Mohammed Abotela, Ahmed Abu-Zaid

**Affiliations:** 1 Department of Medicine, Brigham and Women's Hospital, Boston, USA; 2 Department of Family Medicine, Dr. Sulaiman Al Habib Medical Group, Riyadh, SAU; 3 College of Medicine, Alfaisal University, Riyadh, SAU; 4 College of Medicine, King Abdulaziz University, Rabigh, SAU; 5 College of Medicine, Vision Colleges, Riyadh, SAU; 6 College of Medicine and Medical Science, Arabian Gulf University, Manama, BHR; 7 Faculty of Medicine, Alexandria University, Alexandria, EGY

**Keywords:** national inpatient sample, gallbladder cancer, cholecystitis, gallstones, biliary diseases, helicobacter pylori

## Abstract

Background: *Helicobacter pylori* (*H. pylori*) infection is widely recognized for its association with gastric diseases. Prior studies on the relationship between *H. pylori* infection and biliary diseases have faced constraints, including inadequate control of confounding factors and small sample sizes. This study aims to explore the association between *H. pylori* infection and biliary diseases using a large, population-based sample with adequate control for various covariates.

Methods: The National Inpatient Sample (NIS) from 2016 to 2020 was used to investigate the association between *H. pylori* infection and biliary diseases. We identified patients with *H. pylori* infection using the International Classification of Diseases, Tenth Revision (ICD-10) code (B96.81). Descriptive analysis and inferential statistics, including univariate and multivariate regression, were performed to explore the relationship between *H. pylori* and selected biliary diseases.

Results: Overall, 32,966,720 patients were analyzed. Among them, 736,585 patients had biliary diseases (n=1,637 with *H. pylori* and n=734,948 without *H. pylori*). The baseline characteristics revealed notable differences in demographics and healthcare variables between both groups. Univariate regression analysis demonstrated significant associations between *H. pylori* infection and various biliary diseases such as gallbladder stones, gallbladder cancer, cholangitis, acute cholecystitis, and biliary pancreatitis, with the highest risk for chronic cholecystitis (odds ratio: 5.21; 95% confidence interval: 4.1-6.62; p<0.0001). Multivariate regression analysis, after adjusting for various covariates, confirmed these associations, providing insights into the potential causal relationship between *H. pylori* and biliary diseases.

Conclusion: This study strengthens the evidence suggesting a potential association between *H. pylori* infection and biliary diseases. The findings need to be validated in prospective clinical studies.

## Introduction

*Helicobacter pylori* (*H. pylori*) is a type of gram-negative, mobile, microaerophilic curved bacterium capable of infecting humans through various routes, including oral-oral, gastro-oral, and fecal-oral transmission. This bacterium demonstrates a remarkable ability to thrive in the deep gastric mucus layer. Central to its colonization of the gastric environment is the urease enzyme, which plays a vital role in enabling the bacterium to survive in the harsh, acidic conditions of the stomach, where pH values are typically very low [1]. According to a recent systematic review and meta-analysis, the projected global prevalence of *H. pylori* infection stands at 50% [2].

*H. pylori* has been implicated in a range of both benign and malignant gastric disorders. It is strongly correlated with conditions such as gastric atrophy, peptic ulcer disease, and gastritis, with prevalence rates reported at 44.3%, 20%, and 90%, respectively [3-5]. Additionally, *H. pylori*-related intestinal metaplasia and gastric cancer have rough prevalence rates of 10% and 85%, respectively [4,6,7]. Furthermore, *H. pylori* is frequently detected in patients diagnosed with gastric mucosa-associated lymphoid tissue lymphoma [6,8].

Common biliary conditions embracing gallstones, cholecystitis, and choledocholithiasis have been linked to *H. pylori* infection [9]. Lim et al. in their systematic review highlighted the connection between *H. pylori* and biliary tract diseases, impacting their clinical management [10]. The authors pinpointed that *H. pylori* infection was correlated with cholelithiasis (41.5%) and biliary tract cancer (66.7%), although no association was found with gallbladder polyps [10]. In their systematic review involving 2,932 participants, Wang et al. identified a significant positive correlation between gallbladder infection with *H. pylori* and an elevated risk of cholelithiasis and chronic cholecystitis [9].

Prior reviews and studies on the relationship between *H. pylori* infection and biliary tract diseases have faced constraints, including inadequate control of confounding factors and small sample sizes that may not adequately represent the broader population [6,9,10]. Furthermore, certain studies have failed to identify any association [11]. Given the increased mortality and morbidity risk associated with biliary diseases, understanding the relationship between *H. pylori* and biliary tract disorders is crucial for effective management [10].

Databases sourced from national population data play a critical role in advancing our comprehension of correlational studies. They fulfill a vital function by overcoming limitations stemming from insufficient clinical data and the lack of robust regression analyses. Herein, we investigate the correlation between *H. pylori* infection and biliary tract diseases utilizing extensive, nationally representative data from the United States National Inpatient Sample (NIS) [12]. The results of our investigation offer insights into demographic trends, clinical features, and notable relationships between *H. pylori* and various common biliary pathologies. Additionally, we assess the impact of this association on in-hospital mortality rates and hospital stays. The aim of these findings is to furnish clinical practitioners with valuable data for formulating preventive and therapeutic strategies for biliary diseases in the context of *H. pylori* infection.

## Materials and methods

Our dataset was sourced from the NIS covering the period from 2016 to 2020. The NIS is an extensive database offered by the Agency for Healthcare Research and Quality in the United States [12]. Our specific focus was on individuals hospitalized with a diagnosis of *H. pylori* infection and investigating potential association with various biliary tract disorders, such as chronic cholecystitis, gallbladder stones, gallbladder cancer, cholangitis, acute cholecystitis, and biliary pancreatitis. These conditions were identified using the International Classification of Diseases, Tenth Revision (ICD-10) system (Table 1). To enhance the precision of our analysis, we excluded patients with missing data. Rigorous data cleaning procedures were meticulously carried out using Stata Statistical Software: Release 18 (2023; StataCorp LLC, College Station, Texas, United States).

**Table 1:** The ICD-10 codes and NIS demographics used in the analysis NIS: National Inpatient Sample; ICD-10: International Classification of Diseases, Tenth Revision

Variable	Source	ICD-10 code
Helicobacter pylori	I10_DX1/40	B96.81
Gallbladder stone (cholelithiasis)	I10_DX1/40	K80
Gallbladder cancer	I10_DX1/40	C23
Chronic cholecystitis	I10_DX1/40	K81.1
Acute cholecystitis	I10_DX1/40	K81.0
Cholangitis	I10_DX1/40	K83.0
Cholangiocarcinoma	I10_DX1/40	C22.1
Biliary acute pancreatitis	I10_DX1/40	K85.1
Lipidemia-related disorders	I10_DX1/40	E78.1, E78.2, E.78.3, E78.4, E78.5
Hypertension	I10_DX1/40	I10, I11, I12, I13, I15, I16
Diabetes mellitus	I10_DX1/40	E08, E09, E10, E11, E13
Smoking (tobacco use)	I10_DX1/40	Z72.0
Alcohol misuse	I10_DX1/40	F10.1
Chronic nonsteroidal anti-inflammatory use	I10_DX1/40	Z79.1
Body mass index, underweight (=19.9)	I10_DX1/40	Z68.1
Body mass index, normal weight (20-24.9)	I10_DX1/40	Z68.20, Z68.21, Z68.22, Z68.23, Z68.24
Body mass index, overweight (25-29.9)	I10_DX1/40	Z68.25, Z68.26, Z68.27, Z68.28, Z68.29
Body mass index, obese class 1 (30-34.9)	I10_DX1/40	Z68.30, Z68.31, Z68.32, Z68.33, Z68.34
Body mass index, obese class 2 (35-39.9)	I10_DX1/40	Z68.35, Z68.36, Z68.37, Z68.38, Z68.39
Body mass index, obese class 3 (≥40)	I10_DX1/40	Z68.4
Age	NIS Core	-
Sex	NIS Core	-
Primary expected payer	NIS Core	-
Race	NIS Core	-
Year	NIS Core	-
ZIP income quartile	NIS Core	-
Hospital bed size	NIS Hospital	-
Location/teaching status of hospital	NIS Hospital	-
Hospital region	NIS Hospital	-

In conducting our descriptive analysis, we scrutinized baseline characteristics such as age, sex, body mass index, race, primary payer, median household income (categorized into quartiles), smoking, alcohol misuse, diabetes mellitus, hypertension, lipidemia-related disorders, and chronic nonsteroidal anti-inflammatory use. Moreover, several hospital-related information was collected, including hospital region, hospital bed size, hospital location, and hospital teaching status.

The reporting of categorical data was summarized as numbers and percentages, while numerical data were presented as mean±standard deviation or median (interquartile range). The Shapiro-Wilk test was used to examine the normality of distribution. The chi-squared test was used to explore potentially significant differences between groups with and without *H. pylori* infection. The Mann-Whitney U test was employed to compare means of numerical variables across the groups.

Moving beyond descriptive analysis, inferential statistics were leveraged through univariate and multivariate regression analyses to investigate the connection of *H. pylori* infection in the development of biliary tract diseases. In the multivariate regression model, adjustments for possible covariates were made, incorporating baseline characteristics and pertinent clinical parameters identified in the existing literature. Data were summarized as odds ratios (OR) with corresponding 95% confidence intervals (CI).

Due to the publicly available nature of the database and de-identified data, ethical approval was not required.

## Results

Overall, 32,966,720 patients were analyzed, of whom 736,585 and 32,230,135 patients were diagnosed with and without biliary diseases, respectively. The baseline characteristics of all patients with biliary diseases (n=736,585) based on the presence (n=1,637) or absence (n=734,948) of *H. pylori* diagnosis are displayed in Table 2. Among patients with *H. pylori* diagnosis, 58.16% comprised female patients versus 55.88% in non-*H. pylori* patients. Notably, there was a distinct difference in racial composition, with a lower proportion of White patients in *H. pylori* cases (39.16%) compared to those without *H. pylori* (63.95%). Another noteworthy observation was the higher representation of Hispanic and Black individuals in the *H. pylori* group (29.38% and 29.38%, respectively) as opposed to the non-*H. pylori* group (16.74% and 11.73%, respectively). Medicaid coverage was more prevalent in the *H. pylori* group (25.96%) than in the non-*H. pylori* group (16.93%). Significant differences were also evident in the income quartile distribution; in the *H. pylori* group, a higher percentage fell within the first to 25th quartile (42.09%) compared to the non-*H. pylori* group (29.79%).

**Table 2:** The baseline characteristics of the analyzed biliary disease patients without and with H. pylori infection Numerical data were presented as median (interquartile range), whereas categorical data were presented as number of cases (percentages). Statistical significance was determined at p<0.05. NSAID: nonsteroidal anti-inflammatory drug

Variable	No *H. pylori* diagnosis (n=734,948)	*H. pylori* diagnosis (n=1,637)	P-value
Age (in years)	63 (47-75)	58 (42-70)	<0.0001
Sex			0.065
Male	324,223 (44.12%)	685 (41.84%)	
Female	410,725 (55.88%)	952 (58.16%)	
Primary expected payer			<0.0001
Medicare	355,315 (48.35%)	584 (35.68%)	
Medicaid	124,436 (16.93%)	425 (25.96%)	
Private insurance	194,539 (26.47%)	417 (25.47%)	
Self-pay	391,49 (5.33%)	144 (8.80%)	
No charge	3,599 (0.49%)	13 (0.79%)	
Others	17,910 (2.44%)	54 (3.30%)	
Race			<0.0001
White	469,996 (63.95%)	641 (39.16%)	
Black	86,177 (11.73%)	299 (18.27%)	
Hispanic	123,028 (16.74%)	481 (29.38%)	
Asian or Pacific Islander	25,810 (3.51%)	106 (6.48%)	
Native American	5,292 (0.72%)	22 (1.34%)	
Others	24,645 (3.35%)	88 (5.38%)	
Calendar year			0.774
2016	146,159 (19.89%)	319 (19.49%)	
2017	147,638 (20.09%)	347 (21.20%)	
2018	149,335 (20.32%)	324 (19.79%)	
2019	151,669 (20.64%)	328 (20.04%)	
2020	140,147 (19.07%)	319 (19.49%)	
Median household income quartile			<0.0001
1st-25th	218,930 (29.79%)	689 (42.09%)	
26th-50th	192,161 (26.15%)	361 (22.05%)	
51st-75th	176,692 (24.04%)	333 (20.34%)	
76th-100th	147,165 (20.02%)	254 (15.52%)	
Bed size of hospital			0.001
Small	149,806 (20.38%)	306 (18.69%)	
Medium	219,509 (29.87%)	438 (26.76%)	
Large	365,633 (49.75%)	893 (54.55%)	
Location/teaching status of hospital			<0.0001
Rural	54,208 (7.38%)	158 (9.65%)	
Urban nonteaching	168,017 (22.86%)	316 (19.30%)	
Urban teaching	512,723 (69.76%)	1,163 (71.04%)	
Region of hospital			<0.0001
Northeast	131,033 (17.83%)	283 (17.29%)	
Midwest or North Central	145,145 (19.75%)	255 (15.58%)	
South	285,699 (38.87%)	665 (40.62%)	
West	173,071 (23.55%)	434 (26.51%)	
Diabetes mellitus	204,749 (27.86%)	454 (27.73%)	0.872
Hypertension	427,502 (58.17%)	866 (52.90%)	<0.0001
Lipidemia-related disorders	247,842 (33.72%)	473 (28.89%)	<0.0001
Chronic NSAID use	621,6 (1.04%)	33 (2.49%)	<0.0001
Body mass index (in kg/m^2^)			<0.0001
Underweight (≤19.9)	16,554 (2.25%)	56 (3.42%)	
Normal weight (20-24.9)	14705 (2.00%)	57 (3.48%)	
Overweight (25-29.9)	18760 (2.55%)	54 (3.30%)	
Obese Class 1 (30-34.9)	39885 (5.43%)	96 (5.86%)	
Obese Class 2 (35-39.9)	34762 (4.73%)	84 (5.13%)	
Obese class 3 (≥40)	57525 (7.83%)	107 (6.54%)	
Length of hospitalization (in days)	4 (2-6)	4 (3-8)	<0.0001

Table 3 delineates a significant univariate association between *H. pylori* and all biliary diseases included in our study. Notably, the association between *H. pylori* infection and chronic cholecystitis emerged as the highest correlation (OR: 5.47; 95%CI: 4.42, 6.78; p<0.0001), whereas the association between *H. pylori* infection and cholangitis emerged as the lowest (OR: 1.95; 95%CI: 1.55, 2.44; p<0.0001). Furthermore, significant associations were observed between *H. pylori* and gallbladder cancer (OR: 3.34; 95%CI: 2.18, 5.12; p<0.0001), gallbladder stones (OR: 2.79; 95%CI: 2.64, 2.95; p<0.0001), acute cholecystitis (OR: 2.20; 95%CI: 1.83, 2.64; p<0.0001), biliary pancreatitis (OR: 2.28; 95%CI: 1.83, 2.64; p<0.0001), and cholangiocarcinoma (OR: 2.00; 95%CI: 1.44, 2.77; p<0.0001). Even after adjustment for various confounding factors during multivariate logistic regression analyses, the associations between *H. pylori* and all our selected biliary diseases remained statistically significant (Table 3). 

**Table 3:** Logistic regression analysis for the association between H. pylori diagnosis and biliary diseases Multivariate analysis was adjusted for age, sex, race, hospital region, hospital teaching status, hospital bed size, ZIP income, smoking status, alcohol overuse, diabetes mellitus, lipidemia-related disorders, hypertension, chronic nonsteroidal anti-inflammatory drug use, and body mass index. Statistical significance after Bonferroni correction was determined at p<0.0042. CI: confidence interval

Biliary disease	Univariate analysis		Multivariate analysis		
	Odds ratio (95%CI)	P-value	Odds ratio (95%CI)	P-value	
Gallbladder stone	2.79 (2.64, 2.95)	<0.0001	2.35 (2.21, 2.50)	<0.0001	
Gallbladder cancer	3.34 (2.18, 5.12)	<0.0001	2.15 (1.21, 3.64)	0.004	
Chronic cholecystitis	5.47 (4.42, 6.78)	<0.0001	4.84 (3.81, 6.14)	<0.0001	
Cholangitis	1.95 (1.55, 2.44)	<0.0001	1.70 (1.33, 2.17)	<0.0001	
Biliary pancreatitis	2.28 (1.95, 2.67)	<0.0001	2.00 (1.68, 2.38)	<0.0001	
Acute cholecystitis	2.20 (1.83, 2.64)	<0.0001	1.91 (1.57, 2.34)	<0.0001	
Cholangiocarcinoma	2.00 (1.44, 2.77)	<0.0001	1.50 (1.04, 2.18)	0.032	

## Discussion

Our analysis revealed significant associations between *H. pylori* infection and various biliary diseases. Out of 736,585 admission records for biliary diseases, 1,637 (0.22%) had concurrent *H. pylori* infection. The study found notable demographic differences between patients with and without *H. pylori* infection, including a higher representation of Hispanic/African individuals, and those with Medicaid coverage in the *H. pylori* group. Univariate and multivariate regression analyses showed significant associations between *H. pylori* infection and biliary diseases, with chronic cholecystitis exhibiting the highest correlation. Adjusted associations were also observed for gallbladder stones, gallbladder cancer, cholangitis, acute cholecystitis, and biliary pancreatitis. These findings suggest a potential causal relationship between *H. pylori* infection and biliary diseases.

Our baseline characteristics data revealed several notable trends. For example, *H. pylori* infection and biliary tract diseases were more prevalent among females and individuals of White race, indicating potential demographic patterns. Moreover, there was a higher incidence among those with lower socioeconomic status and those enrolled in Medicare, underscoring socioeconomic disparities in disease burden.  Research has indicated the presence of racial and ethnic discrepancies, revealing that African American and Hispanic communities exhibit a greater frequency of *H. pylori* infection compared to their White counterparts [13]. Likewise, areas characterized by impoverished living conditions and individuals facing economic hardship demonstrate a heightened occurrence of gallstone disease and other biliary disorders, highlighting the significant impact of socioeconomic elements on disease prevalence [14,15]. These revelations emphasize the imperative of confronting systemic racism, poverty, and inadequate healthcare accessibility to mitigate the disproportionate burden of *H. pylori* infection and biliary disease across diverse demographic and socioeconomic strata.

Our regression analysis highlights a robust association between *H. pylori* infection and chronic cholecystitis, gallbladder stones, gallbladder cancer, cholangitis, acute cholecystitis, and biliary pancreatitis, suggesting a broad impact on various biliary diseases, which is consistent with previous literature [4,6-9,16-19]. Notably, chronic cholecystitis depicted the strongest association with *H. pylori* infection. Chronic cholecystitis is mostly caused by gallbladder stones, and some potential risk factors for gallbladder stones include obesity, female gender, increasing age, rapid weight loss, pregnancy, hormone replacement therapy, cholesterol-lowering drugs, and certain clinical disorders such as diabetes mellitus and liver disease [20]. Herein, our research suggests that *H. pylori *infection could be an equally important risk factor for the development of gallstones and chronic cholecystitis. A pooled analysis of 26 prospective studies revealed that smoking seems to elevate the risk of developing all biliary tract cancers (except for gallbladder cancer) and that alcohol consumption may heighten the risk of cholangiocarcinoma [21].

From a pathophysiological perspective, for *H. pylori* bacterium to cause infection within the biliary tract, two basic requirements must be met, namely, the capability to reach the intended site and the ability to thrive in that environment. Although primarily a resident of the gastric mucosa, *H. pylori* has been detected in the biliary system, suggesting potential translocation and, consequently, the potential for disease-causing effects. Current prevailing theories propose that *H. pylori* may gain access to bile either through retrograde reflux from the sphincter of Oddi or hematogeneously from the portal circulation [22]. Once *H. pylori* translocates to the biliary system, it endures the relatively alkaline environment via the reflux of bile from the duodenum into the stomach, contributing to the selection of specific *H. pylori* strains that are tolerant to bile salts [23]. Alternatively, inflammation resulting from biliary pathologies may inadvertently lower biliary pH, creating a more favorable environment for *H. pylori* [24]. This underscores the capacity of *H. pylori*'s ability to access and persist within the biliary tract. Such capability provides both the avenue and the mechanism for *H. pylori*, a recognized group 1 carcinogen, to induce chronic inflammation and malignancy in the hepatobiliary system [10]. Mechanistically, *H. pylori* triggers multiple oncogenic, intracellular pathways within epithelial cells, including phosphoinositide-3-kinase (PI3K), nuclear factor kappa B (NF-κB), and Wnt/β-catenin [25-27]. These pathways influence various cellular functions, leading to the heightened production of inflammatory cytokines, altered apoptosis rates, and enhanced epithelial cell proliferation and differentiation, ultimately culminating in the oncogenic transformation of biliary epithelial cells. Many of these effects are attributed to *H. pylori* virulence factors such as cytotoxin-associated antigen A (CagA), the cag pathogenicity island (PAI), vacuolating cytotoxin (VacA), and outer membrane proteins (OMPs) [28].

Eradication of *H. pylori* may help prevent or manage biliary diseases. For example, Zhang et al. found a statistically significant reduction in gallstone prevalence among *H. pylori*-eradicated patients compared to no prior eradication [16]. Hence, several studies have proposed that implementing a "test and treat" protocol for *H. pylori* infection could potentially decrease the prevalence of biliary diseases and associated complications, including cancer [7,29]. By identifying and treating *H. pylori* infection, clinicians may be able to mitigate the aftermath of certain biliary conditions in susceptible individuals.

This study leverages data from the NIS, which is a comprehensive database covering a significant proportion of hospitalizations in the United States. The large sample size and representative nature of the dataset enhance the generalizability and reliability of the study findings. The study employs rigorous data cleaning procedures and statistical analyses to ensure the accuracy and robustness of the results. By meticulously examining baseline characteristics and conducting both univariate and multivariate regression analyses, the study provides a comprehensive assessment of the link between *H. pylori* infection and biliary tract disorders. Compared with previous literature, this study investigates a wide range of biliary pathologies, including chronic cholecystitis, gallbladder stones, gallbladder cancer, cholangitis, acute cholecystitis, and biliary pancreatitis. By examining multiple outcomes, the study offers a more comprehensive understanding of the potential influence of *H. pylori* infection on biliary health. The study identifies several demographic patterns linked to *H. pylori* infection and biliary diseases, including sex, race, socioeconomic status, and insurance coverage, just to name a few. By elucidating these patterns, the study contributes valuable insights into the epidemiology of *H. pylori*-related biliary diseases and highlights potential areas for targeted interventions and further research. 

However, this study harbors several shortcomings. Firstly, it is retrospective and observational, which introduces the possibility of potential confounding factors affecting the findings. Secondly, there is a lack of clarity regarding the extent to which patients underwent testing for *H. pylori* infection or the specific diagnostic criteria used for making the diagnosis. It is plausible that some diagnoses were based solely on clinical evaluation rather than laboratory testing. This is significant because, without testing, it is difficult to ascertain whether patients without a diagnosis of *H. pylori* infection truly did not have the condition. Thirdly, the nature of the NIS dataset means that the same patients may appear multiple times within a given year or across different years, as individual patients cannot be identified. Additionally, there may be inaccuracies in the use of ICD-10 codes for biliary diseases across hospitals. Lastly, the temporality of *H. pylori* infection and the development of biliary tract disease cannot be ascertained.

The present findings have significant implications for clinical practice, guiding healthcare providers in the management and prevention of biliary diseases associated with *H. pylori* infection. Furthermore, by identifying gaps in current knowledge and suggesting areas for future research, the study lays the foundation for further investigations aimed at elucidating the underlying mechanisms and temporal relationship of the association between *H. pylori* infection and biliary diseases. To thoroughly examine the association between *H. pylori* infection and biliary disease, further prospective studies with extended follow-up periods are warranted. These studies would provide valuable insights into the long-term implications of *H. pylori* infection on the development and progression of various biliary pathologies. Besides, researchers can better assess the temporal relationship between *H. pylori* infection and the onset or exacerbation of biliary disorders. Moreover, such studies can enable the evaluation of outcomes such as disease recurrence, progression, or the development of complications. They also can help address potential confounding factors and biases that may influence the observed associations. By controlling relevant variables and utilizing rigorous study designs, researchers can enhance the validity and reliability of their findings.

## Conclusions

Our study strengthens the evidence suggesting a potential link between *H. pylori* infection and various biliary tract disorders. Analysis of nationally representative United States NIS data reveals significant links, highlighting the need for ongoing investigation. Our findings stress the importance of considering various factors in understanding disease etiology. Moving forward, prospective studies are crucial for a clearer understanding and exploring preventive and therapeutic strategies.
